# Neutrophil extracellular traps and monocyte subsets at the culprit lesion site of myocardial infarction patients

**DOI:** 10.1038/s41598-019-52671-y

**Published:** 2019-11-08

**Authors:** Andreas Mangold, Thomas M. Hofbauer, Anna S. Ondracek, Tyler Artner, Thomas Scherz, Walter S. Speidl, Konstantin A. Krychtiuk, Roela Sadushi-Kolici, Johannes Jakowitsch, Irene M. Lang

**Affiliations:** Department of Internal Medicine II, Division of Cardiology, Medical University of Vienna, Vienna, Austria

**Keywords:** Mechanisms of disease, Neutrophils, Monocytes and macrophages, Myocardial infarction

## Abstract

Neutrophils release their chromatin into the extracellular space upon activation. These web-like structures are called neutrophil extracellular traps (NETs) and have potent prothrombotic and proinflammatory properties. In ST-elevation myocardial infarction (STEMI), NETs correlate with increased infarct size. The interplay of neutrophils and monocytes impacts cardiac remodeling. Monocyte subsets are classified as classical, intermediate and non-classical monocytes. In the present study, *in vitro* stimulation with NETs led to an increase of intermediate monocytes and reduced expression of CX3CR1 in all subsets. Intermediate monocytes have been associated with poor outcome, while non-classical CX3CR1-positive monocytes could have reparative function after STEMI. We characterized monocyte subsets and NET markers at the culprit lesion site of STEMI patients (n = 91). NET surrogate markers were increased and correlated with larger infarct size and with fewer non-classical monocytes. Intermediate and especially non-classical monocytes were increased at the culprit site compared to the femoral site. Low CX3CR1 expression of monocytes correlated with high NET markers and increased infarct size. In this translational system, causality cannot be proven. However, our data suggest that NETs interfere with monocytic differentiation and receptor expression, presumably promoting a subset shift at the culprit lesion site. Reduced monocyte CX3CR1 expression may compromise myocardial salvage.

## Introduction

Myocardial infarction (MI) and subsequent ischemic cardiomyopathy are a major disease burden worldwide^1^. The pathomechanism of coronary thrombosis, ischemia and myocardial scar formation is incompletely understood. We have previously shown that neutrophils release neutrophil extracellular traps (NETs) at the culprit lesion site (culprit site), which might serve as scaffolds for platelets and erythrocytes and could initiate coronary thrombosis^2^, especially in the setting of superficial plaque erosion^3^. Coronary NET burden significantly correlated with reduced ST-segment resolution and increased infarct size in ST-elevation MI (STEMI) patients. Degradation of NETs by deoxyribonucleases in circulation is crucial for homeostasis^4^. High endogenous deoxyribonuclease activity was correlated with reduced infarct size^2^. Hence, increased NET burden due to impaired degradation appears to be detrimental downstream in the ischemic myocardium, causing microvascular obstruction, inflammation and cardiomyocyte cell death^5^. NET-releasing neutrophils and monocytes cooperate in inflammation^6^, thrombus formation^7^ and cardiac remodeling^8^. Monocytes can differentiate into macrophages^9^, which release major proinflammatory mediators like Interleukin-1ß in response to NETs^10^. Macrophages in turn complement the clearance of NETs^11^ and are shifted towards an anti-inflammatory phenotype after phagocytosis of neutrophilic proteins^12^. Monocyte subsets have specific functions in cardiac remodeling after MI^13^ and are grouped according to their CD14/CD16 expression into classical (CD14++ CD16−), intermediate (CD14++ CD16+) and non-classical (CD14+ CD16++) monocytes^14^. A recent study has shown that monocyte subsets evolve from classical to intermediate and non-classical monocytes^15^. Classical monocytes are proinflammatory and produce high levels of cytokines^16^. Intermediate monocytes are potent antigen-presenting cells, which is reflected by a high HLA-DR expression^17^. They are proinflammatory^18^, exhibit a strong phagocytic activity and strongly contribute to oxidative burst. Non-classical monocytes display the highest CX3CR1 (fractalkine receptor) expression, which is an important receptor for adhesion and migration. They have a patrolling function and efficiently invade inflamed tissue. Via CX3CR1, they respond to apoptotic signals to remove cell debris and contribute to tissue repair, thus exerting anti-inflammatory properties^19^. Classical and intermediate monocytes are increased in the periphery in the course of MI and correlate with increased infarct size and poor cardiac function at follow up^20,21^, whereas non-classical monocytes were associated with improved recovery of dysfunctional myocardium^22^.

In the present study, we investigated the impact of NETs on monocyte subsets *in vitro* and evaluated NET surrogate markers and monocytes subsets *in vivo* at the culprit site of STEMI patients.

## Methods

### Patients

STEMI patients (n = 91), who received thrombectomy in the course of primary percutaneous coronary intervention (pPCI) at the Department of Cardiology, General Hospital of Vienna, were consecutively included in the study and monocyte subsets were quantified in femoral and culprit site blood samples. An additional characterization of monocyte expression markers was performed in the last 36 consecutive patients. Inclusion criteria were chest pain at the time of pPCI, ST-elevations of ≥2 mm on >1 chest lead or new ST-elevations of ≥1 mm on >1 limb lead within 20 minutes of pPCI. All patients received 250 mg of acetylsalicylic acid and unfractionated heparin to achieve an activated coagulation time of >300 seconds (4000–10000 IE). Criteria for thrombectomy were a vessel diameter of ≥3 mm, a large intraluminal contrast medium filling defect, suggestive of thrombus within 50 mm of the respective ostium without severe calcification, tortuosity, or difficult vascular access. A total volume of 10 to 20 mL culprit site blood were aspirated using a commercial thrombectomy catheter (Pronto [Vascular Solutions], Export [Medtronic], Diver [Invatec], and Thrombuster [Atrium Medical Corporation]). Femoral whole blood samples were obtained simultaneously from the femoral arterial sheath. All plasma measurements were normalized for femoral whole blood hematocrit as aspiration catheter flushing with 2–4 ml 0.9% sodium chloride leads to dilution of the culprit site blood sample. Exclusion criteria were low culprit site sample volume, intake of immunosuppressants, cardiogenic shock, severe infection, active neoplasia, end stage kidney disease and glycoprotein IIb/IIIa-blocker treatment. Infarct size was estimated by measuring creatine phosphokinase isoform MB area under the curve (CK-MB AUC), which allows an adequate approximation of infarct size (Supplementary Fig. 1).

All study participants gave written informed consent prior to the inclusion under an approval of the Ethics Committee of the Medical University of Vienna, Austria (approval reference number 303/2005). All investigations conformed to the principles outlined in the Declaration of Helsinki.

### Flow cytometry

Blood samples were incubated with fluorochrome-labeled antibodies for 30 minutes in the dark. Cells were stained for human CD11a (PE, Biolegend), CD11b (PE, Biolegend), CD14 (FITC, Biolegend), CD16 (PE-Cy7, Biolegend), CD45 (APC-Cy7, Biolegend), CD56 (APC, Biolegend), CD66b (PerCP-Cy5.5, Biolegend), CD142 (PE, BD Pharmingen) CD192 (PE, Biolegend), toll-like receptor (TLR)2 (PE, Biolegend), TLR4 (PE, Biolegend), CX3CR1 (PE, Biolegend) and HLA-DR (PE, Biolegend). Erythrocyte lysis by BD FACS lysing solution (BD Biosciences) was followed by 3 washing steps with phosphate-buffered saline (PBS), then cells were resuspended for the measurements. Per panel, 50000 events were counted and the percentage of positive cells or mean fluorescence intensity (MFI) was measured.

Monocyte subsets were gated as depicted in Supplementary Fig. 2. Cells were separated from debris and doublets; CD45+ CD66b− cells were divided from lymphocytes by forward and side scatter gating, then CD56− monocytes were divided into the subsets according to their CD14/CD16 expression profile. In preliminary experiments, viability of cells was assessed using annexin V / propidium iodide FC staining technique (Apoptosis detection kit, BD Biosciences); Viability of gated monocytes was >98% (data not shown).

Isotype-matched antibodies (mouse IgG1, IgG2a and IgM, Biolegend) were added to negative control samples. Cells were analyzed using a BD FACSCanto II and FACSDiva Software (BD Biosciences).

Total monocyte subset numbers were calculated via monocyte numbers measured in femoral and culprit site differential cell counts similar to a published approach^23^. Culprit site differential blood counts could be obtained from 69 patients.

### NET surrogate markers

Citrullinated histone H3 (citH3) was measured as described^24^, with minor modifications. Streptavidin-coated plates were incubated with anti-histone biotin (Roche) for 120 minutes. Plates were washed and incubated with plasma samples or standard (citH3, Cayman) for 90 minutes. After washing, anti-histone H3 (1:2000 in PBS + 1% bovine serum albumin [BSA], Abcam) was added for 60 minutes. Plates were washed and incubated with goat anti-rabbit HRP conjugate antibody (1:5000 in PBS + 1% BSA, BioRad) for 60 minutes, washed, and developed with BM Blue POD Substrate (Roche) for 20 minutes. The reaction was stopped by addition of 2 M H_2_SO_4_ and optical density was measured on a Promega GloMax Discover microplate reader (450 nm, reference 620 nm).

Double-stranded deoxyribonucleic acid (dsDNA) was quantified using the *Quant-iT PicoGreen dsDNA Assay* (Invitrogen) according to manufacturer´s instructions. Fluorescence was measured on a GloMax Discover microplate reader (Promega).

### CX3CL1 measurements

CX3CL1 was quantified using the *Human CX3CL1/Fractalkine Quantikine ELISA Kit* (R&D Systems) according to manufacturer’s instructions in the same subset of patients, from whom an expression marker analysis was performed by flow cytometry (FC) (n = 34). Optical density was measured on a Promega GloMax Discover microplate reader (450 nm, reference 620 nm).

### Isolation of neutrophil extracellular traps

Neutrophils and NETs were isolated from healthy donors according to recently published protocols with minor modifications^25,26^. Purity of neutrophils was routinely above 98%. Neutrophils were resuspended in RPMI 1640 (Gibco) + 3% fetal calf serum (FCS, Gibco) and stimulated with 500 nM phorbol-myristate acetate for 4 hours at 37 °C. Supernatant was discarded, and cells were incubated with RPMI containing 3% FCS and 10 U/ml Alu I (Roche) for 30 minutes at 37 °C. Supernatant was collected and centrifuged for 5 minutes, 300 g to remove remaining cellular debris. Concentration of NETs was assessed using PicoGreen as described above. A detailed analysis of NETs produced *in vitro* can be found in Supplementary Fig. 3.

### Stimulation of monocytes with NETs *in vitro* in whole blood cultures

50 µl of EDTA whole blood drawn from healthy volunteers were stimulated with 500 ng/ml isolated NETs or vehicle control in a polystyrene tube for 1 hour at 37 °C. Blood was washed with 2 ml PBS + 1% FCS. Supernatant was discarded, and the remaining pellet was resuspended in 50 µl PBS. Cells were stained for CD66b (Pacific Blue), CD45 (Pacific Orange), CD142 (Brilliant Violet 711), CD14 (FITC), CX3CR1 (PE), HLA-DR; CD16 (PE-Cy7), CD56 (Alexa Fluor 647), CD11b (Alexa Fluor 700), HLA-DR (APC-Cy7). Cells were lysed using BD FC Lysis Solution for 15 minutes. Samples were washed three times with PBS + 1% FCS and analyzed using an Attune NxT Flow Cytometer (ThermoFisher).

### Statistics

Distribution of data was assessed by histograms complemented by Kolmogorov-Smirnov test and Shapiro-Wilk test. Non-parametric data are given as median and interquartile range [IQR] and analyzed using Wilcoxon signed rank test and Spearman rank correlation (r_s_). Normally distributed data are given as mean ± standard deviation (SD) and tested using paired Students t-test. Bonferroni-Holm correction was applied in each set of experiments to correct for multiple testing. Boxplots display the 25th–75th percentile, the whiskers cover the 5th–95th percentile. Extreme outliers (<5th or >95th percentile) are not displayed in the figures. A published trapezoidal formula was used to calculate CK-MB AUC^27^, with at least 5 consecutive values over a period of 3 days after pPCI. A p-value below 0.05 was considered significant. Statistical testing was performed using IBM SPSS Statistics 22.0 for Windows.

## Results

### Patient characteristics

We included 91 patients suffering from STEMI with a TIMI flow 0–1 who underwent pPCI including thrombectomy at the Vienna General Hospital. Mean age was 57.2 ± 13.0 years, 19.8% were women. Detailed patient characteristics are provided in Table 1.

**Table 1:** Patient characteristics.

Patient characteristics (n = 91)		
Age, years		57.2 ± 13.0
Female sex, n (%)		18 (19.8)
Diabetes, n (%)		9 (9.9)
History of hypertension, n (%)		55 (60.4)
Ever smokers, n (%)		66 (72.5)
Family history of CAD, n (%)		34 (37.4)
BMI > 30 kg/m², n (%)		33 (36.3)
Culprit vessel, n (%)	LAD	40 (44.0)
	CX	15 (16.5)
	RCA	36 (39.6)
	Multiple culprit lesions	3 (2.7)
CAD, n (%)	1VD	56 (61.5)
	2VD	18 (19.8)
	3VD	17 (18.7)
CRP, nmol/l	<*48*	45.7 [21.9–94.2]
CK-MB max, U/l	<24	226 ± 239
TnT, µg/l	<*0.03*	2.6 ± 2.8
Creatinine, µmol/l	*50–100*	97.2 ± 35.4
Cholesterol, mmol/l	<*5.2*	5.15 ± 1.12
LDL, mmol/l	<*4.1*	3.02 [2.16–3.87]
HDL, mmol/l	>*1.5*	1.09 ± 0.22
Triglycerides, mmol/l	<*1.7*	1.4 [1.0–2.61]
Final TIMI flow grade		2.7 ± 0.7
Symptom-to-balloon time, min		246 [160–372]

Data are presented as number (percent) of patients, mean ± SD or median [IQR]. Normal ranges are in the central column in italic. CAD, coronary artery disease; BMI, body mass index; LAD, left anterior descending artery; CX, left circumflex artery; RCA, right coronary artery; VD, vessel disease; CRP, C-reactive protein; CK-MB max, maximal creatine phosphokinase isoform MB; TnT, troponin T; LDL, low density lipoprotein cholesterol; HDL, high density lipoprotein cholesterol.

### Stimulation of monocytes with NETs *in vitro* shifts classical towards intermediate monocytes and strongly influences receptor expression

**Figure 1:**
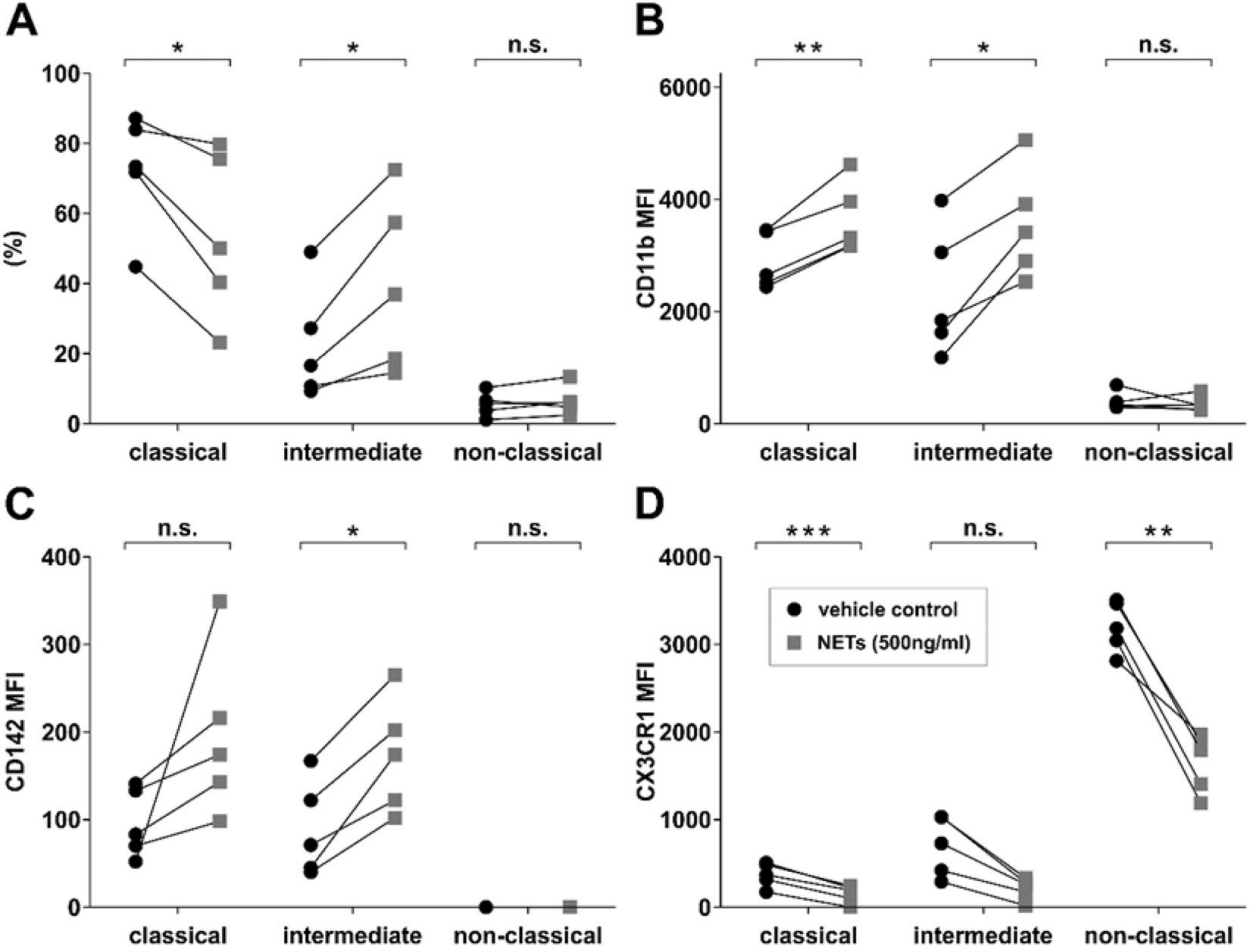
Stimulation of monocytes with NETs *in vitro*. (**A**) Monocyte subset shift as % of total monoctes, expression of (**B**) CD11b, (**C**) CD142 and (**D**) CX3CR1 of classical, intermediate and non-classical monocytes. Monocyte subsets in percent (%) or mean fluorescence intensity (MFI) levels are displayed. Monocytes were stimulated in whole blood from healthy donors (n = 5) with NETs derived from isolated neutrophils from a healthy donor (n = 1) or vehicle control for 60 minutes. *p < 0.05, **p < 0.01, ***p < 0.001.

Whole blood samples from healthy donors (n = 5) were stimulated with isolated NETs *in vitro* and monocyte subsets were measured by FC. Stimulation with NETs led to a shift from classical to intermediate monocytes (Fig. 1A). Moreover, an increased expression of CD11b on classical and intermediate monocytes, an increased expression of CD142 only on intermediate monocytes, but a decreased expression of CX3CR1 on all three subsets was detected (Fig. 1B–D). Stimulation of monocytes from a single donor with isolated NETs from 5 different donors showed the same expression pattern (Supplementary Fig. 4).

### NET surrogate markers are increased at the culprit lesion site and correlate with myocardial injury

**Figure 2:**
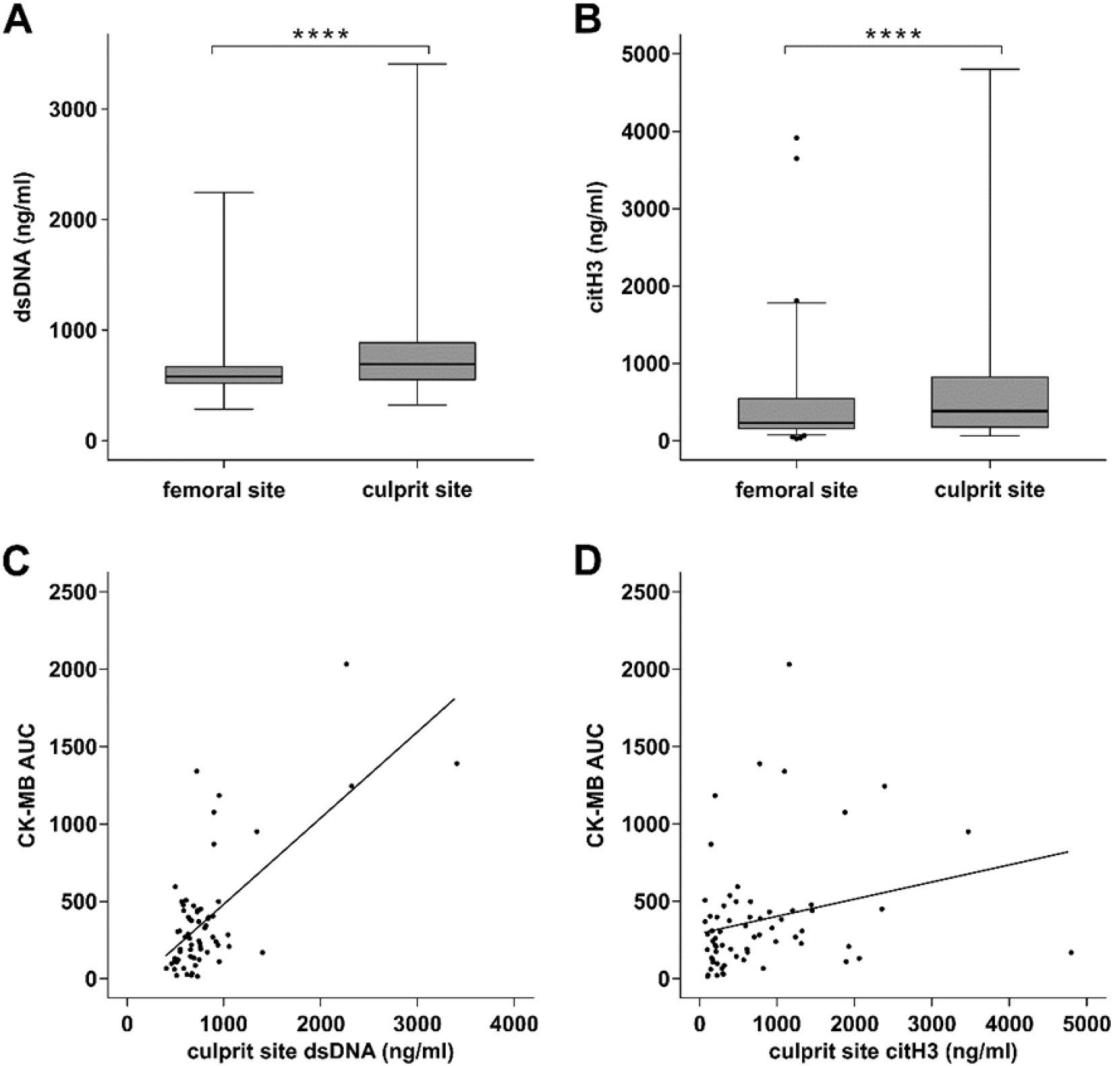
Surrogate markers of neutrophil extracellular traps. (**A**) Double-stranded DNA (dsDNA, n = 83, femoral 581.7 [519.8–672.7] versus culprit site 692.7 [552.7–888.0] ng/ml) and (**B**) citrullinated histone 3 levels (citH3, n = 87, femoral 233 [156.5–545.6] versus culprit site 380.5 [173.8–820.3] ng/ml) were significantly increased at the culprit site of STEMI patients. Enzymatic infarct size expressed as the creatine phosphokinase isoform MB area under the curve (CK-MB AUC) correlated with (**C**) double-stranded DNA (dsDNA, n = 65, r_s_ = 0.409, p < 0.001) and (**D**) citrullinated histone 3 (citH3, n = 65, r_s_ = 0.315, p < 0.01). Significance ****p < 0.0001.

In line with previous data^2,28^, dsDNA was significantly increased at the culprit site compared to femoral plasma (Fig. 2A). Citrullinated histone 3 was also substantially increased in culprit site plasma compared to femoral plasma (Fig. 2B). Furthermore, dsDNA (Fig. 2C) and citH3 (Fig. 2D) levels correlated with enzymatic infarct size measured as CK-MB AUC.

### Monocyte subsets are shifted at the culprit lesion site of STEMI patients

**Figure 3:**
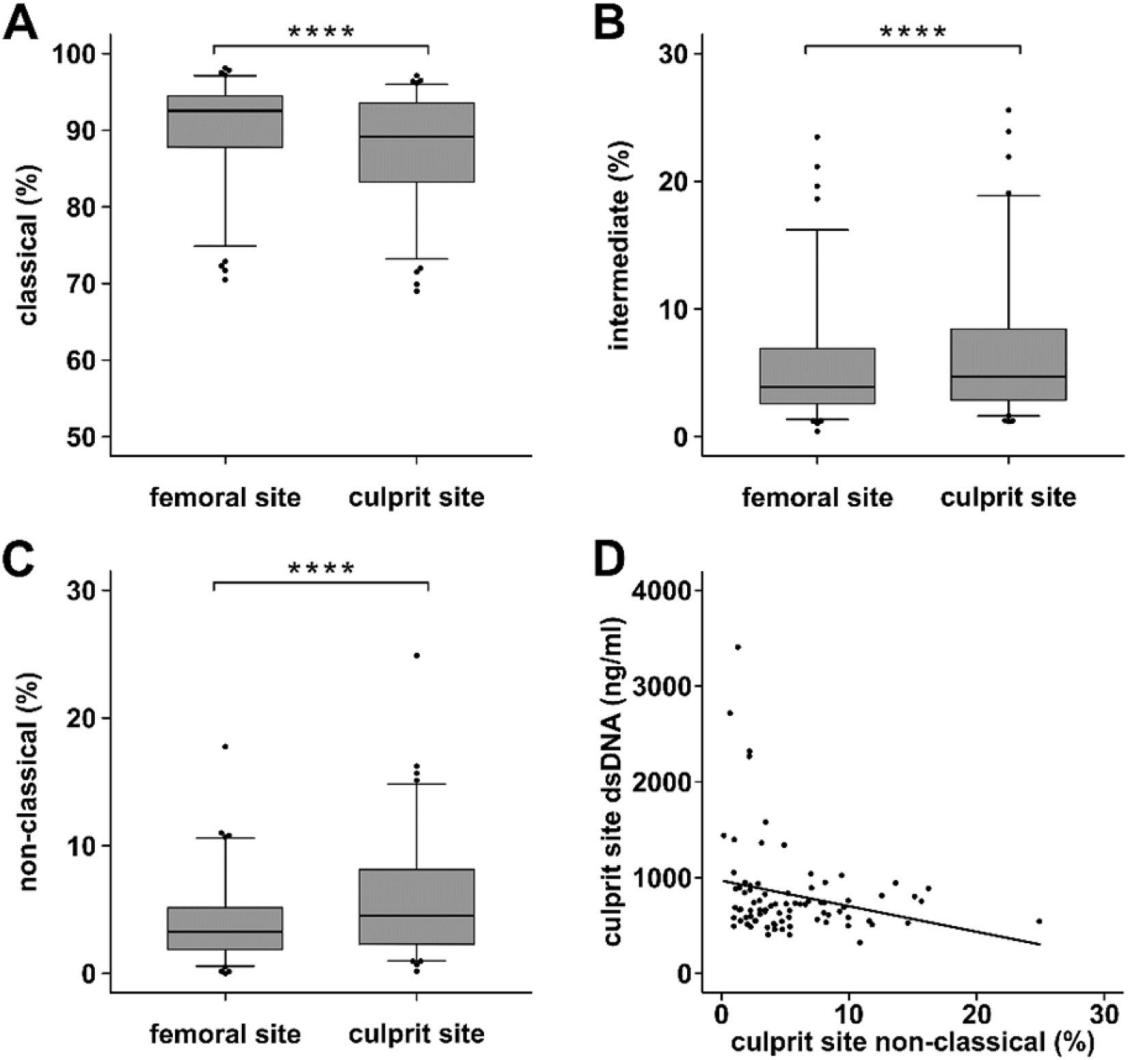
Monocyte subsets at the culprit lesion site of STEMI patients. (**A**) Classical monocytes (femoral 91.4 [85.2–94.0] versus culprit site 89.2 [82.8–92.9] %), (**B**) intermediate monocytes (femoral 4.5 [2.9–7.9] versus culprit site 5.2 [3.1–8.9] %) and (**C**) non-classical monocytes (femoral 3.6 [2.0–7.19] versus culprit site 4.9 [2.5–8.2] %) were determined by flow cytometry as % of total monoctes in femoral and culprit site whole blood samples of STEMI patients (n = 91). The gating strategy is depicted in Supplementary Fig. 2. (**D**) Culprit site dsDNA correlates with non-classical monocyte percentage (n = 63, r_s_ = −0.224, p < 0.05). ****p < 0.0001.

Monocytes subsets were determined in culprit site and femoral whole blood from STEMI patients (n = 91). Monocyte percentage of leukocytes was significantly decreased in culprit site whole blood compared to the femoral site (femoral 3.7 [2.6–5.1] versus culprit site 3.1 [2.3–4.1] %, p < 0.0001), but total monocyte number in differential blood cell counts was not significantly different (femoral 600 [500–800] versus culprit site 600 [400–800] cells/l, p = not significant). Interestingly, classical monocyte percentage was decreased at the culprit site (Fig. 3A), whereas intermediate (Fig. 3B) and non-classical monocyte percentages were increased (Fig. 3C). High culprit site dsDNA correlated with low non-classical monocyte percentage at the culprit site (Fig. 3D). Culprit site citH3 related to non-classical monocyte numbers accordingly, but was not statistically significant.

In total cell numbers, intermediate (n = 69, femoral 23 [14–54] versus culprit site 29 [15–50] cells/l, p < 0.05) and non-classical monocytes (n = 69, femoral 22 [10–38] versus culprit site 26 [18–44] cells/l, p < 0.0001) were significantly increased, while classical monocytes were not significantly different (n = 69, femoral 580 [426–739] versus culprit site 547 [394–731] cells/l, p = not significant).

### Monocyte subsets display differentially expressed activation markers at the culprit lesion site

**Figure 4:**
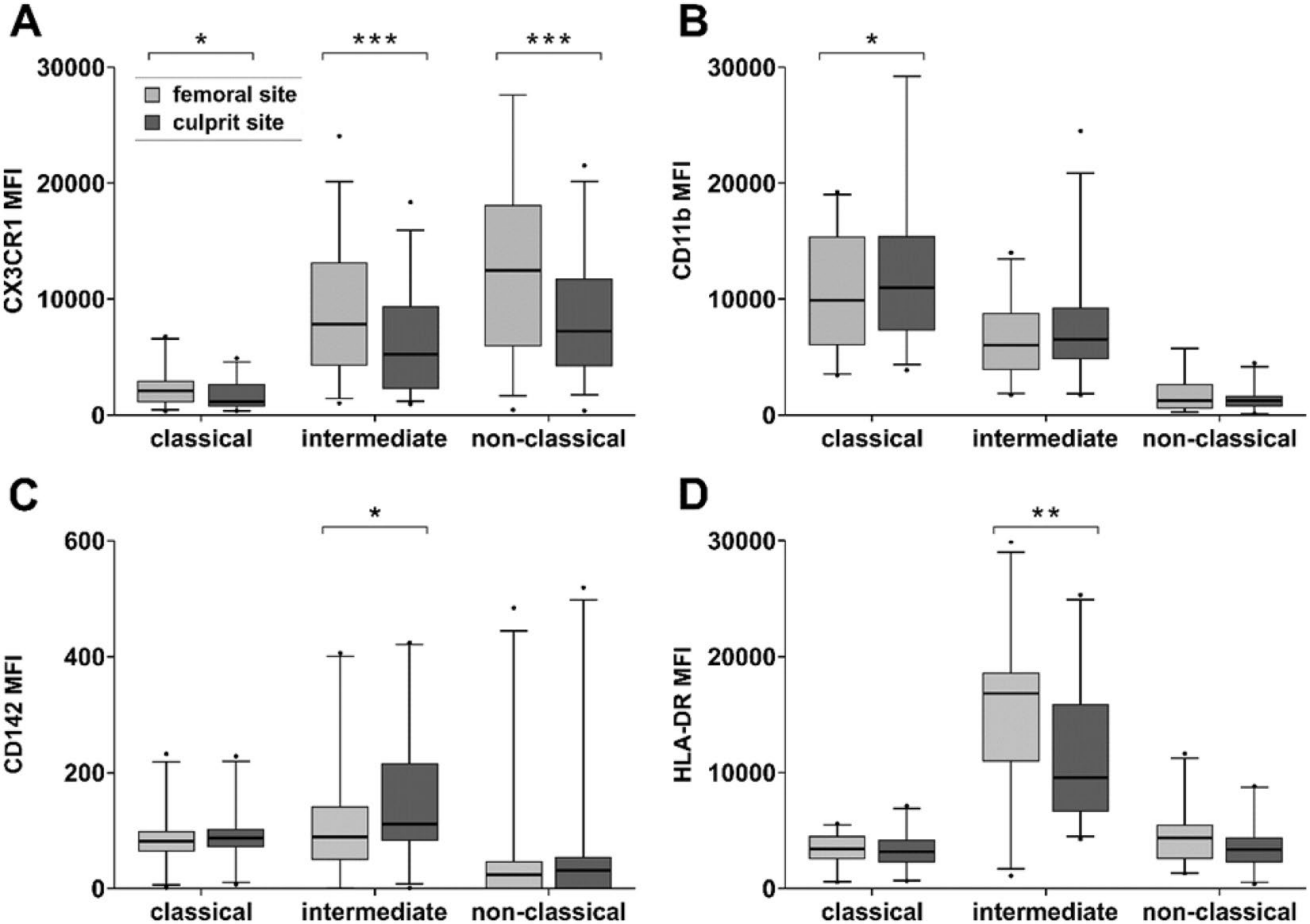
Expression markers of monocyte subsets in STEMI patients. Expression markers were determined by flow cytometry in femoral and culprit site whole blood samples (n = 36). (**A**) CX3CR1, (**B**) CD11b, (**C**) CD142 and (**D**) HLA-DR on classical monocytes, intermediate monocytes and non-classical monocytes. Data are presented as mean fluorescence intensity (MFI) of monocyte subsets. All expression marker data are listed in Supplementary Table 1. *p < 0.05, **p < 0.01, ***p < 0.001.

We determined surface receptors of monocytes at the culprit site compared to the femoral site by FC in a subset of patients (n = 36). As expected, classical monocytes displayed a constitutively low expression of CX3CR1, while intermediate and especially non-classical monocytes showed distinctly higher levels. The CX3CR1 expression was substantially decreased at the culprit site in all subsets (Fig. 4A).

Soluble CX3CL1 was measured in the same patients and was significantly decreased in culprit site compared to femoral plasma (n = 32, femoral site 620 ± 280 pg/ml versus culprit site 540 ± 300 pg/ml, p < 0.01).

Classical monocytes displayed increased CD11b expression at the culprit site compared to the femoral site (Fig. 4B), CD142 expression of intermediate monocytes was significantly increased at the culprit site (Fig. 4C), while HLA-DR expression was decreased (Fig. 4D). Other measured activation markers (CD11a, CD192, TLR2, TLR4) were not significantly different between the femoral site and the culprit site. All FC data are listed in Supplementary Table 1.

### Low monocytic CX3CR1 expression is connected to higher NET burden and increased myocardial injury

**Figure 5:**
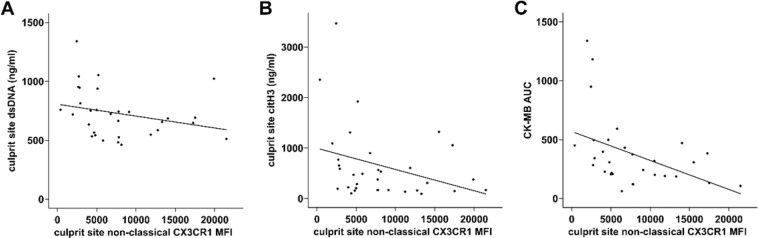
Correlation of non-classical monocyte CX3CR1 expression with NET surrogate markers and infarct size. Low expression of CX3CR1 on non-classical monocytes correlates with higher (**A**) double-stranded DNA (dsDNA, n = 31, r_s_ = −0.423, p < 0.05), (**B**) citrullinated histone 3 (citH3, n = 33, r_s_ = −0.359, p < 0.05) and (**C**) enzymatic infarct size expressed as the creatine phosphokinase isoform MB area under the curve (CK-MB AUC, n = 30, r_s_ = −0.556, p < 0.01).

Low CX3CR1 expression of non-classical monocytes correlated with high culprit site dsDNA (Fig. 5A) and citH3 levels (Fig. 5B). Interestingly, we detected a strong correlation between increased enzymatic infarct size measured as CK-MB AUC and low expression of CX3CR1 on classical monocytes (n = 30, r_s_ = −0.517, p < 0.05), intermediate monocytes (n = 30, r_s_ = −0.569, p < 0.01) and non-classical monocytes (n = 30, r_s_ = −0.556, p < 0.01, Fig. 5C).

Symptom-to-balloon time did not correlate with monocyte frequencies, expression markers or NETs surrogate markers (n = 33).

## Discussion

In the present study, we investigated monocyte subsets at the culprit site of STEMI patients. In absence of a suitable model for coronary atherothrombosis and lack of an animal model that approximates human STEMI, the local pattern of immune cells at the culprit site of patients is crucial to unravel the pathophysiology of MI. Percentage of monocytes was decreased in culprit site blood compared to the femoral site, which might be explained by the local accumulation of neutrophils^29^. Neutrophils and NETs have a detrimental impact on reperfusion and infarct size in STEMI^2,30^. It has been proposed that NETs are involved in microvascular obstruction and downstream injury^5^. In line with previous data, we detected increased levels of NET surrogate markers in culprit site plasma, which correlated with higher enzymatic infarct size (Fig. 2)^2,28,31^. Of note, NETs are not the exclusive possible source of extracellular DNA and histones, thereby it could be very possible that dying cells are another important source of extracellular chromatin in STEMI patients. Although the exact mechanisms of reperfusion injury and adverse remodeling are unknown, one important component might be the interplay between neutrophils and monocytes^7^. We investigated the influence of NETs on monocyte subsets *in vitro* and detected a significant shift from classical towards intermediate monocytes. Monocyte subsets most likely evolve from classical over intermediate to non-classical monocytes^15^. Intermediate monocytes are associated with features of vulnerable plaques^32^ and were reported to be connected to worse outcome in STEMI^21,33^. Furthermore, we observed a strongly diminished CX3CR1 expression of intermediate and non-classical monocytes, but increased expression of CD11b and CD142 (tissue factor) on classical and intermediate monocytes *in vitro* (Fig. 1). These findings underscore reports on the proinflammatory and prothrombotic effects of extracellular chromatin^34–36^.

We measured the same expression markers at the culprit site of STEMI patients and found a very similar pattern. Intermediate and non-classical monocytes (as percentage of monocytes as well as in total numbers) were significantly increased compared to the femoral site, and higher dsDNA correlated with lower non-classical monocyte levels (Fig. 3). In spite of only partial comparability of human and murine monocyte subsets, *Nahrendorf et al*. proposed an interesting two-step model for myocardial healing after ischemia derived from animal data. An early CCR2-dependent influx of ‘inflammatory’ Ly6c-high monocytes is followed by a CX3CR1-dependent takeover of ‘reparative’ Ly6c-low monocytes^37^. Indeed, it was shown that human CD16-positive monocytes, which most likely correspond to murine Ly6c-low monocytes, remain increased in peripheral blood up to 12 days after pPCI compared to CD16-negative monocytes, which peak after 3 days^20,21^. In autopsy studies, the CD16+ monocyte number in the infarct zone peaked in the proliferative phase after MI^38^. Our data showing non-classical monocyte accumulation at the culprit site indicate that homing to the ischemic myocardium is initiated considerably earlier after coronary occlusion.

High NET surrogate markers correlated with low CX3CR1 expression of non-classical monocytes (Fig. 5) and culprit site CX3CR1 expression was strongly reduced compared to the femoral site (Fig. 4). Yet, high CX3CR1 expression by non-classical monocytes correlated with smaller infarct size (Fig. 5). The CX3CR1 receptor is discussed to enable effective non-classical monocyte trafficking and is critical for resolution of tissue inflammation and healing^19^. It was shown that repair after arterial injury is compromised in CX3CR1 knockout mice and can be rescued by application of wild type Ly6c-low monocytes^39^. Similar results were reported from a model of ischemic kidney reperfusion injury^40^. In contrast, it was shown that CX3CL1 neutralization in a murine myocardial infarction model resulted in improved survival^41^.

Soluble CX3CL1 (fractalkine), the major ligand of CX3CR1, was reported to be increased during STEMI^42^. We found decreased levels of CX3CL1 at the culprit site compared to femoral plasma. Hypoxia led to reduced CX3CL1 release of endothelial cells *in vitro*^43^. Of note, pulmonary endothelial cells responded differently^44^. Receptor internalization after ligand binding could be an alternative explanation for reduced CX3CR1 expression and CX3CL1 at the culprit site, however, our *in vitro* data do not support this hypothesis.

Interestingly, NETs also promote differentiation of monocytes into fibrocytes *in vitro*. *In vivo*, fibrocytes are increased at the culprit site, which appears to also favor adverse tissue remodeling^31^.

Taken together, these data suggest that extracellular chromatin could promote a differentiated monocyte response and lower CX3CR1 expression on monocytes at the culprit site. This possibly leads to decreased transmigration into the ischemic tissue of non-classical monocytes and impaired myocardial healing. Targeting NETs in acute coronary syndrome may be a promising therapeutic strategy.

## Limitations

With the present study, we provide information on monocyte subsets in MI. However, sampling from the culprit and femoral site of STEMI patients only yields a snapshot of ongoing processes. In this human system, causality cannot be established and our findings remain observational. The effect size of presented data from the femoral and culprit site is small, however paired comparisons are from the same circulatory system, therefore also small differences might be very meaningful.

## Supplementary information

Supplementary information

## Data Availability

All data and protocols can be promptly made available.
