# Supplementary material for: Neutrophil extracellular traps and monocyte subsets at the culprit lesion site of myocardial infarction patients
Source: Sci Rep. 2019 Nov 8;9:16304. doi: 10.1038/s41598-019-52671-y (PMC6841683; doi:10.1038/s41598-019-52671-y)
Supplement: Supplementary file 1 — Supplementary information [file 41598_2019_52671_MOESM1_ESM.pdf]

Supplementary information

Neutrophil extracellular traps and monocyte subsets at the culprit lesion site of myocardial infarction patients

Andreas Mangold, Thomas M. Hofbauer, Anna S. Ondracek, Tyler Artner, Thomas Scherz, Walter S. Speidl, Konstantin A. Krychtiuk, Roela Sadushi-Kolici, Johannes Jakowitsch, Irene M. Lang*

Department of Internal Medicine II, Division of Cardiology, Medical University of Vienna

*Address for correspondence: Irene M Lang, MD, Professor of Vascular Biology, Department of Internal Medicine II, Division of Cardiology, Medical University of Vienna, Waehringer Guertel 18-20, 1090 Vienna, Austria, phone: +431 40400 4614, fax: +431 40400 4612, email: irene.lang@meduniwien.ac.at.

Supplementary Table 1

Monocyte subsets		femoral site	culprit site	p value
**Classical monocytes**	CD11a	7262.5 [5826-9772]	8243.5 [6180.5-9705]	ns
	CD11b	9894 [6207-14929]	10963 [7723-15146]	p<0.05
	CD142	81.5 [67-96]	86.5 [73-99]	ns
	HLA-DR	3409 [2595-4492]	3184 [2291-4160]	ns
	CX3CR1	2115.5 [1153.5-2919]	1138 [781-2623.5]	p<0.05
	TLR2	3126 [2406-3966]	3240 [2363-3896]	ns
	TLR4	214 [191-308]	242 [178-282]	ns
	CD192	933.5 [594.5-1410.5]	912 [542.5-1393.5]	ns
**Intermediate monocytes**	CD11a	8642.5 [7274.5-11607.5]	9250.5 [6920.5-11319]	ns
	CD11b	6083 [4323-9420]	6621 [4995-9641]	ns
	CD142	89 [53-139]	111 [84-209]	p<0.05
	HLA-DR	16835 [10995-18600]	9581 [6644-15867]	p<0.01
	CX3CR1	7862 [4464-12887]	5259 [2330-9323.5]	p<0.001
	TLR2	2855 [2495-4006]	3147 [2245-4155]	ns
	TLR4	282 [202-373]	288 [236-357]	ns
	CD192	549 [420.5-930]	630 [418.5-1088]	ns
**Non-classical monocytes**	CD11a	7795 [5771-9353]	7379 [5799-8477.5]	ns
	CD11b	1117.5 [368-2603]	1245.5 [817-1714]	ns
	CD142	23.5 [0-43]	31.5 [0-53]	ns
	HLA-DR	4398 [2626-5548]	3385 [2273-4397	ns
	CX3CR1	12463 [6092.5-17989]	7245.5 [4308-11555]	p<0.001
	TLR2	1166 [987-1544]	954 [881-1302]	ns
	TLR4	91 [35-154]	83 [33-124]	ns
	CD192	340.5 [207-538]	391 [317.5-637.5]	ns

Supplementary Table 1: Activation marker expression of monocyte subsets in STEMI patients. Listed markers were measured on monocyte subsets from the femoral and the culprit site of STEMI patients (n=36) using flow cytometry to determine monocytic activation. Date are given as median [IQR]. Significance was determined by Wilcoxon‘s signed-rank test.

Supplementary Figure 1

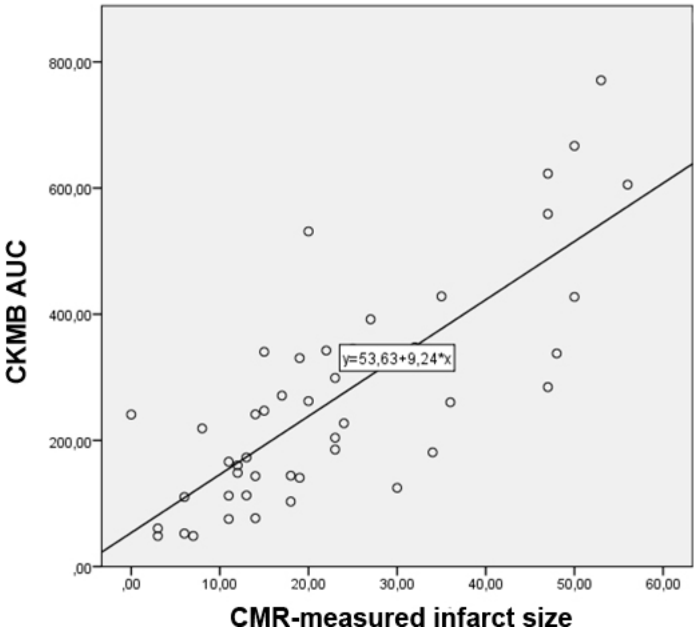

Supplementary Figure 1: Correlation of creatine phosphokinase isoform MB area under the curve (CK-MB AUC) with cardiac magnet resonance (CMR)-measured infarct size. CK-MB AUC was validated in the control group of a prospective, controlled study[1]. A published trapezoidal formula was used to calculate CK-MB AUC[2], with at least 5 consecutive values over a period of 3 days after pPCI. A strong correlation is shown (n=46, r=0.786, p<0.001).

Supplementary Figure 2

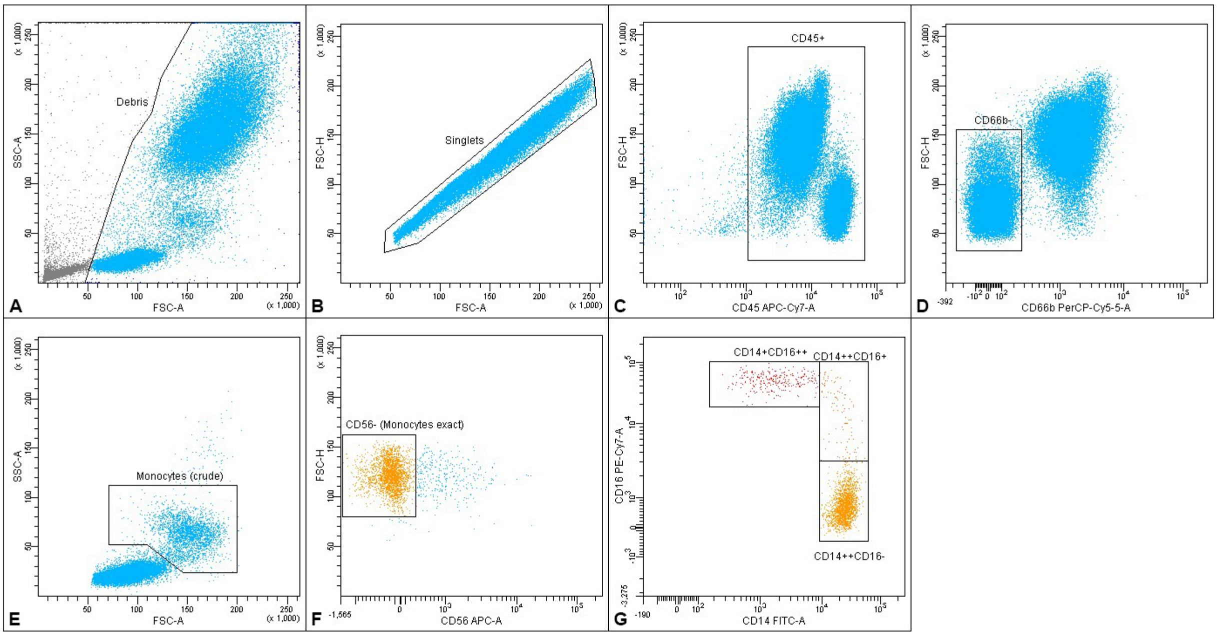

Supplementary Figure 2: Gating strategy for monocyte subsets. Cells were separated from debris (**A**) and doublets (**B**); CD45+CD66b- cells (**C**, **D**) were divided from lymphocytes by forward and sideward scatter gating (**E**), then CD56- monocytes (**F**) were divided into the subsets according to their CD14/CD16 expression profile (**G**).

Supplementary Figure 3

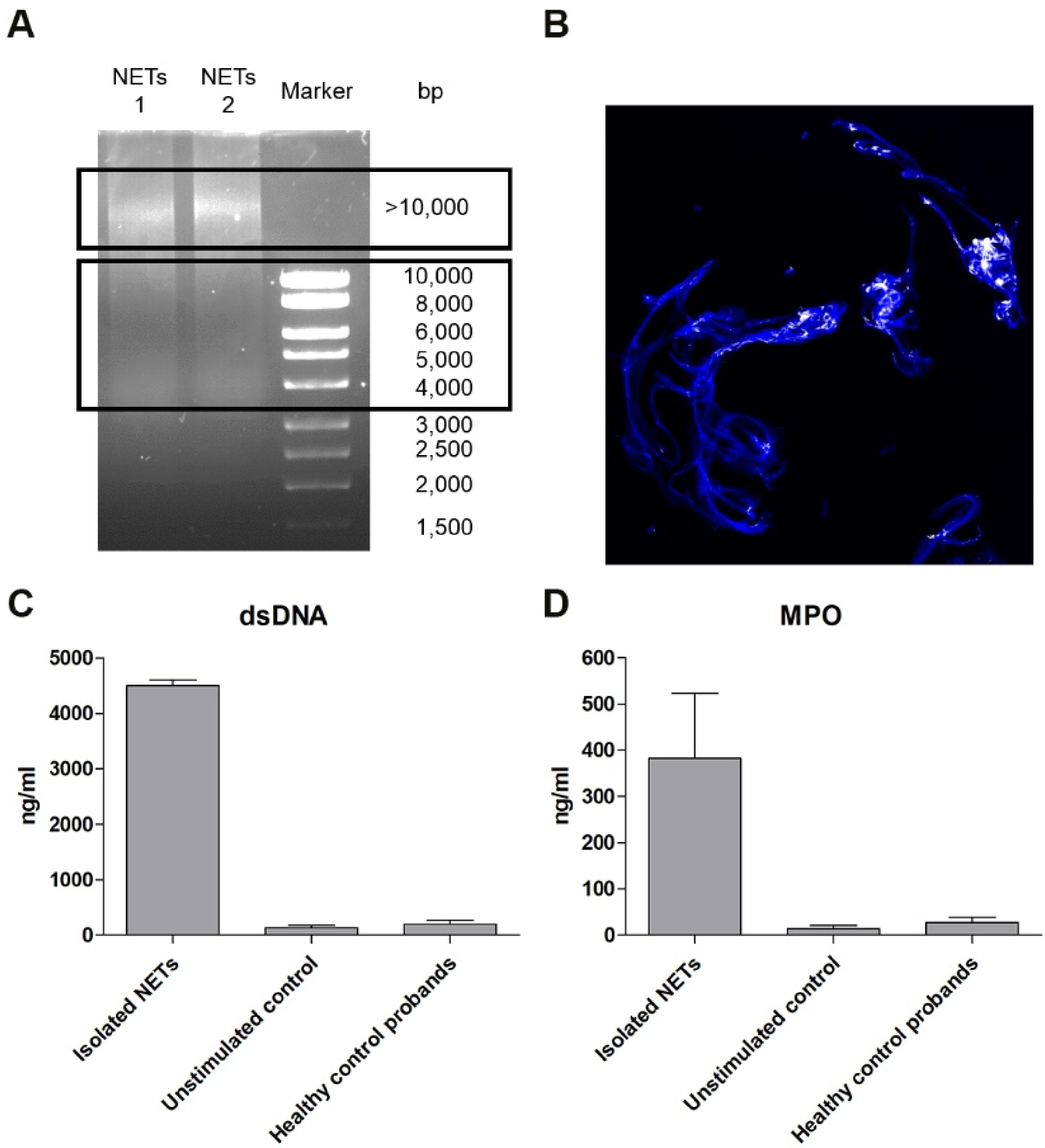

Supplementary Figure 3: Characterization of isolated neutrophil extracellular traps. Neutrophil extracellular traps (NETs) were produced, isolated and processed as described in the main document of the manuscript according to published protocols[3]. The solubilized compound was run in an electrophoresis gel (**A**). As expected, a smear of DNA over a wide range of lengths is visible.

Neutrophils were stimulated *in vitro* in chamber slides and stained utilizing immunofluorescence. Typical strand-like structures can be observed (**B**). DNA was stained using DAPI (blue), myeloperoxidase (MPO) was stained using a rabbit anti-human MPO antibody (Abcam). The slides were scanned utilizing TissueFAXS (TissueGnostics). For the analysis, TissueQuest software (Version 4.01.0128) was used.

The solubilized compound displayed high levels of double-stranded DNA (dsDNA, **C**) and MPO (**D**) compared to unstimulated *in vitro* controls as well as healthy control probands (n=200). Double-stranded DNA was measured as described in the main document of the manuscript. MPO concentration was determined utilizing a commercial Human MPO Instant ELISA (Thermo Fisher). The assay was performed according to the manufacturers' protocol.

Supplementary Figure 4

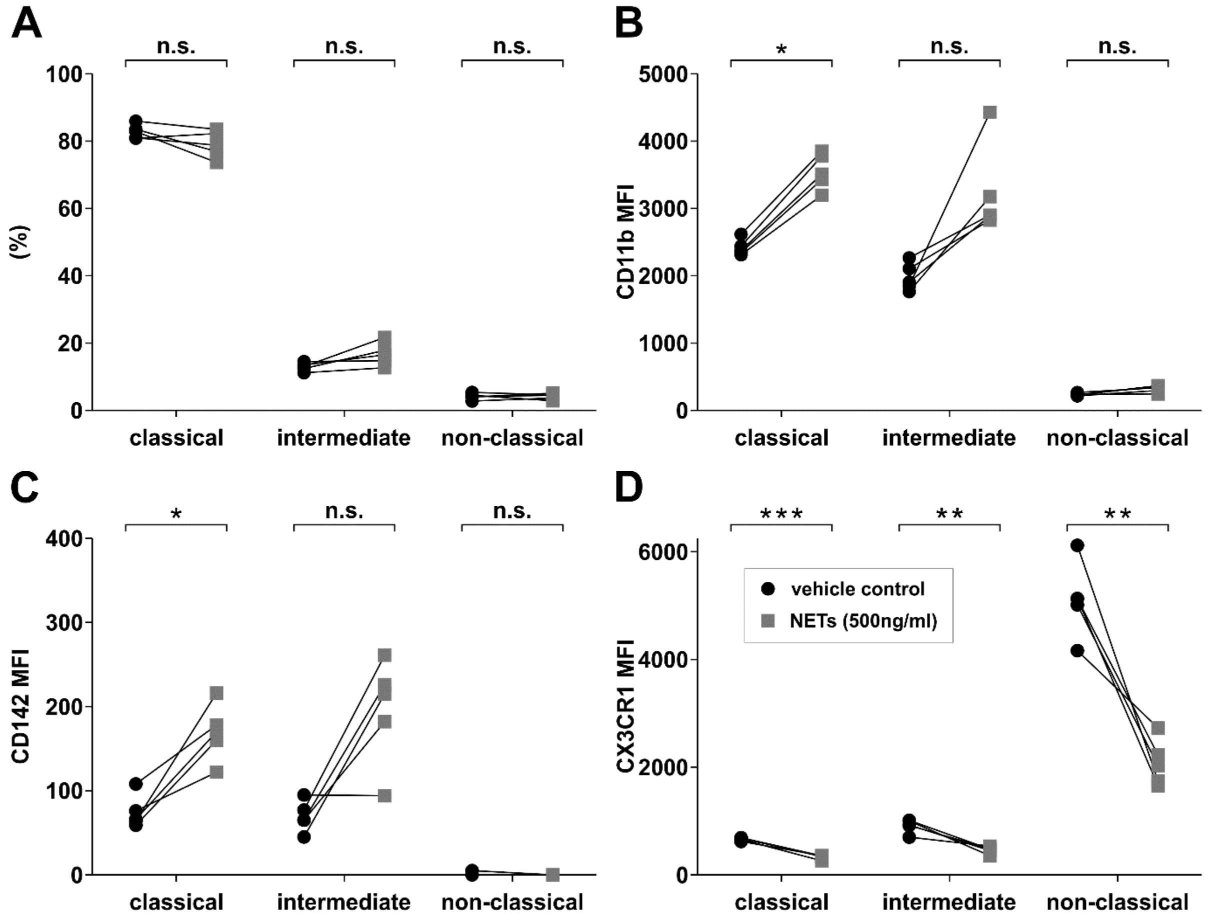

Supplementary Figure 4: Stimulation of monocytes with NETs *in vitro*. Monocytes subset shift (**A**), expression of (**B**) CD11b, (**C**) CD142 and (**D**) CX3CR1 of classical, intermediate and non-classical monocytes from a healthy donor (n=1). Mean fluorescence intensity (MFI) levels or monocyte subsets in percent (%) are displayed. Monocytes were stimulated in whole blood with NETs derived from isolated neutrophils from healthy donors (n=5) or vehicle control for 60 minutes. Significance was determined by Student's paired t-test. *p<0.05, **p<0.01, *** p<0.001.

References

1 Testori, C. *et al.* Out-of-hospital initiation of hypothermia in ST-segment elevation myocardial infarction: a randomised trial. *Heart* **105**, 531-537, doi:10.1136/heartjnl-2018-313705 (2019).

2 Crimi, G. *et al.* Remote ischemic post-conditioning of the lower limb during primary percutaneous coronary intervention safely reduces enzymatic infarct size in anterior myocardial infarction: a randomized controlled trial. *JACC. Cardiovascular interventions* **6**, 1055-1063, doi:10.1016/j.jcin.2013.05.011 (2013).

3 Silvestre-Roig, C. *et al.* Externalized histone H4 orchestrates chronic inflammation by inducing lytic cell death. *Nature* **569**, 236-240, doi:10.1038/s41586-019-1167-6 (2019).
